# Influence of Feeding Quinoa (*Chenopodium quinoa*) Seeds and Prickly Pear Fruit (*Opuntia ficus indica*) Peel on the Immune Response and Resistance to *Aeromonas sobria* Infection in Nile Tilapia (*Oreochromis niloticus*)

**DOI:** 10.3390/ani10122266

**Published:** 2020-12-01

**Authors:** Shaimaa A. A. Ahmed, Ghada I. Abd El-Rahman, Amany Behairy, Rasha R. Beheiry, Basma M. Hendam, Faisal M. Alsubaie, Samah R. Khalil

**Affiliations:** 1 Department of Fish Diseases and Management, Faculty of Veterinary Medicine, Zagazig University, Zagazig 44511, Egypt; shaimaakarim2020@gmail.com; 2 Department of Clinical Pathology, Faculty of Veterinary Medicine, Zagazig University, Zagazig 44511, Egypt; gana660@gmail.com; 3 Department of Physiology, Faculty of Veterinary Medicine, Zagazig University, Zagazig 44511, Egypt; amanybehairy25688@gmail.com; 4 Department of Histology and Cytology, Faculty of Veterinary Medicine, Zagazig University, Zagazig 44511, Egypt; rasharagab2006@yahoo.com; 5 Department of Husbandry and Development of Animal Wealth, Faculty of Veterinary Medicine, Mansoura University, Mansoura 35511, Egypt; d.basma.gentic@gmail.com; 6 Central Veterinary Laboratory, Department of Genome, Riyadh 11454, Saudi Arabia; iamfaisal77@gmail.com; 7 Department of Forensic Medicine and Toxicology, Faculty of Veterinary Medicine, Zagazig University, Zagazig 44511, Egypt

**Keywords:** immunostimulant, natural adjuvants, bacterial infection, interferon-*γ*, transforming growth factor-beta

## Abstract

**Simple Summary:**

The inclusion of dietary supplements as feed additives in fish feed promotes the growth, immunity, and health of the fish, thereby accomplishing extraordinary outcomes in the net gain of the farm. Therefore, the present study was conducted to evaluate the influence of using quinoa seeds (QU) and prickly pear fruit peel (PP) as dietary supplements for fish, at the dose levels of 10% and 20% of the diet, on the immune response and disease resistance against pathogens, providing a novel perspective in aquaculture. Our findings indicated that the inclusion of PP and QU into the diets of Nile tilapia (*Oreochromis niloticus*) as feed supplements improved the survival rate, as well as the hematological, digestive, antioxidant, and immunological parameters. Moreover, an improvement in the strength of Nile tilapia immune response against *Aeromonas sobria* (*A. sobria*) infection was observed, evidenced by the improvement in the survival rate of infected fish. This was accomplished through the protection of the hepatic tissue and modulation of the expression of immune-encoding genes, including the downregulation of the gene encoding *TGF-β* and upregulation of the *IFN-γ*-encoding gene. Moreover, histological restoration of the morphological structures of intestine, liver, and spleen tissues was observed, particularly at the supplementation level of 20%.

**Abstract:**

In recent times, nutraceuticals have been used extensively to identify promising feed additives for the improvement of the aquaculture industry through the enhancement of growth and survival rates, potentiation of the immune responses, and fortification of the resistance against infectious bacterial diseases. In this study, Nile tilapia (*Oreochromis niloticus*) were fed with diets supplemented with quinoa seeds (QU) or prickly pear fruit peel (PP) at the dose levels of 10% or 20% of the diet. After 45 days of the feeding trial, the fish were exposed to *Aeromonas sobria* (*A. sobria*) challenge. The pre-challenge indices indicated that both supplements mediated a significant improvement in most of the estimated parameters, including survival rate, antioxidant status, hematological and immunological indices, and hepatoprotective potential. These effects were recorded in the groups fed with high doses of the supplements (20%). The least changes were observed in the QU_10_-supplemented fish. In the spleen tissue, the *TGF-β* gene was upregulated in the PP_10_-, PP_20_- and QU_20_-supplemented groups, while the expression of the *IFN-γ* gene remained unaffected in all the supplemented groups, except for the PP_20_-supplemented group, which showed an upregulation. After the challenge with *A. sobria*, the relative survival percentage was improved by the supplementation of PP and QU, particularly in the PP_20_-supplemented group, possibly via the promotion of immunological responses, hepatoprotective potency, and modulation of the studied genes. Moreover, the morphological structure of the tissues showed marked recovery. The findings suggest that Nile tilapia fed with different levels of PP peel and QU seeds, particularly at the level of 20%, enhanced the immune response in fish and improved their resistance against *A. sobria* infection.

## 1. Introduction

The inclusion of nutraceuticals in fish feed as supplements aims to ensure the promotion of growth, immunity, and health of the fish to accomplish extraordinary outcomes in the net gain of the farm. Hence, in fish-cultivating systems, feed supplements represent a well-known management strategy, with various supplements exhibiting the ability to enhance the fish immune response or control the severity of various infections affecting the aquatic life [1].

Tilapia (*Oreochromis niloticus*) is among the most popular cultivable fish for freshwater aquaculture due to its high marketing value, growth rate, productivity, and resistance to environmental conditions [2]. It is an economically important fish species and is expected to account for 62% of the total global aquaculture production by 2030 [3]. Proper feeding of a nutritionally balanced diet is critical for the success of any tilapia-farming strategy. In order to produce high growth rates, tilapia fish are typically fed with moderate to high protein diets at rates ranging from 1% to 30% of their body weight/day, depending upon the age and size of the fish and whether it accepts artificial feeding since the hatching stage [4]. During the production of tilapia, large mass mortality could be caused by several bacterial infections, particularly those by the *Aeromonas* species that widely affect various freshwater fish species under the conditions of stress [5]. The pure protein with fatty material composition of the fish body is a major factor contributing to the high vulnerability of various fish to bacterial infections, as these substrates favor bacterial growth [6]. Moreover, the water in which fish live is a favorable medium for the growth of different bacterial strains [7]. *Aeromonas* spp. are predominant inhabitants of the aquatic environment and the gastrointestinal tracts of healthy fish. Under stress conditions, these bacteria become pathogenic, causing septicemia, hemorrhage, and ulcerative fish diseases, thereby leading to mass mortalities among the affected fish and high economic losses in the aquaculture sector [8]. *Aeromonas sobria* (*A. sobria*) have been reported to affect various farmed and wild fish species, such as tilapia (*Oreochromis* sp.), perch (*Perca fluviatilis*), walking catfish (*Clarias batrachus*), common carp (*Cyprinus carpio*), *rainbow trout*, wild-spawning gizzard shad, (*Dorosoma cepedianum*), and mud loach (*Misgurnus mizolepis*) [9,10]. The clinical signs of the disease caused by *A. sobria* infection in fish include ulcerative dermatitis, tail and fin rot, and septicemia [11]. *A. sobria* can infect other vertebrates, including humans, causing severe localized and systemic infections in immunocompromised people [12].

The European Union has prohibited the use of antibiotics in aquaculture due to the development of several drug-resistant bacterial strains and their residues, which accumulate in fish bodies and aquatic ecosystems [13]. Moreover, the high costs of immunostimulants make them uneconomical for fish farming [14]. Hence, the introduction of natural immunostimulants as feed additives is a crucial alternative and eco-friendly strategy for disease control and treatment [15]. Recently, medicinal plants have been utilized for supplementing health and immunity and positively impacting the growth performance by conferring efficient protection against oxidative injury and related disease [16].

Among these medicinal plants, quinoa (*Chenopodium quinoa*), a pseudocereal or pseudo-grain, is a member of the plant family Amaranthaceae. Recently, interest in its seed consumption has increased progressively due to its highly-valuable nutritional composition, making it a potential novel functional food. It is rich in a variety of macronutrients and micronutrients [17]. These seeds have also been found to contain various bioactive components, including carotenoids, vitamin C, flavonoids, phytosterols, and polyphenolic compounds with potential nutraceutical benefits. These compounds contribute to several physiological properties, such as protection against several diseases, including allergy and cancer, and may also lower the risk of cardiovascular diseases. Moreover, the antimicrobial, antioxidant, and anti-inflammatory activities make quinoa (QU) seeds valuable functional food [18,19]. The antioxidant potential of QU has been attributed to the phenolic and flavonoid compounds, which play important roles in inhibiting the free radicals, protecting from oxidative stress, and promoting health [20,21]. Few studies have evaluated the antimicrobial activities of QU seeds [22,23]. The inclusion of QU plant in the feed of cattle, pigs, and poultry livestock, as a rich nutritional source, has been reported [24]. Jacobsen et al. [25] concluded that the dietary inclusion of 150 gm QU per kg diet for broiler chickens has a positive effect on growth performance. However, little information on the nutraceutical potential of QU seeds in aquatic animals is available.

During the processing of fruits, large quantities of byproducts, including peels of fruits, are generated, which are eventually discarded. With proper utilization, these byproducts could serve as a potential novel source of bioactive functional compounds [26]. Prickly pear plant (PP) (*Opuntia ficus indica* L.), a member of the family Cactaceae, is cultivated widely in Latin South Africa, America, and the Mediterranean region [27]. Several studies on PP have focused on the fruits, which have excellent nutritional and medicinal properties [28].

Although the peel of the PP fruit represents about 30–60% of the total weight of the entire fruit, with previous reports describing it as an excellent source of bioactive components and a few studies conducted on its beneficial uses [29,30]. Betalains, a polyphenolic compound of PP, possesses anti-inflammatory, antioxidant, antimicrobiological, anti-lipidemic, and anticancer properties. Betalains, along with other polyphenols constituents of PP, play an important role in the prevention of certain diseases [31,32]. The PP fruits have several pharmacological functions, such as the regulation of immunity and protection of gastric functions in the gastrointestinal tract [33,34], due to which the use of Opuntia peels is recommended as food or functional-food supplements to promote the health of humans and animals. Moreover, the PP cladodes and mesocarps maintain the viability of sheep lymphocytes by hindering the production of H_2_O_2_, thus reducing oxidative injury and increase the production performances of farm animals by improving their immunity [35].

Several critical markers, including hematological profile, serum biochemistry, and immune response-linked variables, can be utilized for monitoring the health status in response to the inclusion of dietary feed additives and numerous immunomodulatory agents in Nile tilapia aquaculture. The immunological variables such as lysozyme, myeloperoxidase (MPO), and nitric oxide (NO) are the major components of the innate immunity of fish. They possess the ability to destroy pathogens via the lysis of the bacterial cell wall, promotion of the inflammatory process, and killing-off the invasive microorganisms [36]. The expression patterns of immune-related genes have been analyzed to decipher the influence of these genes on the strength of the immune response at the transcriptional level. Interferon-γ (IFN-γ) has an important role in regulating the innate and adaptive immune responses against intracellular bacterial infections. It stimulates the macrophage-mediated phagocytosis, regulates the pro-inflammatory cytokines, induces proteins that bind to iron, and creates a bacteriostatic environment by limiting the availability of iron to the pathogens [37]. Prabu et al. [38] reported a notable increase in the expression level of the IFN-γ gene after *A. hydrophila* infection. Moreover, transforming growth factor-β (TGF-β) is important for the immune response. It performs a dual role in regulatory T cells (Treg) and pro-inflammatory T cells, producing interleukin-17 (Th17), which leads to cell differentiation [39]. Previous studies by Awad et al. [40] have shown that some immunostimulants and probiotics can increase the levels of TGF-β1 expression in rainbow trout (*Oncorhynchus mykiss*). Moreover, histopathological investigation is widely acknowledged as a biological marker to explore the pathological changes resulting from various biological infectious agents [41]. Furthermore, the host-resistance challenge assay is a definitive test for assessing the immune system response with the highest degree of biological relevance since it validates the integrated immune response at the whole organism level [42].

The present study was conducted to determine the influence of the dietary supplementation of QU seeds and PP fruit peels, at two concentrations, on the immune status of Nile tilapia. This was achieved by assessing the growth performance and the physiological, digestive, and immunological parameters. Moreover, the expression pattern of the immune-encoding genes, the associated tissue-structure changes, and the susceptibility to *A. sobria* infection were also evaluated.

## 2. Materials and Methods

### 2.1. Fish and Rearing Conditions

Two hundred and twenty-five healthy Nile tilapia fingerlings (21–25 g) were obtained from El-Abbassa Fish Hatchery, Al-Sharkia Province, Egypt. The fingerlings were randomly subjected to routine clinical, parasitological, and bacteriological examination for the presence of pathological lesions, infestations, and infections, respectively. Then, they were acclimatized for 14 days in 160 L glass aquaria filled with dechlorinated tap water and fed with basal diet twice daily at a rate of 5% of the fish biomass per day, without the addition of the tested supplements (Table S1) [43]. The water quality parameters were adjusted according to the guidelines of the American Public Health Association [44]. All the glass aquaria in the laboratory were subjected to the same conditions of pH (6.9 ± 0.1), temperature (25 ± 1.02 °C), dissolved oxygen (7.4 ± 0.34 mg/L), and ammonia (0.035 ± 0.01 mg/L), with a controlled photoperiod of 10 h light: 14-h dark. The bottom third of the water was changed daily to discard excretory wastes. All the experimental procedures and the methodology were approved by the Institutional Animal Care and Use Committee of Zagazig University (ZU-IAURC) (Approval No., ZU-IACUC/2 F/124/2020).

### 2.2. Formulation of Tested Diets

PP fruits were obtained from the local market of Zagazig city, Al-Sharkia Province, Egypt. The fruits were thoroughly washed and peeled within a day. The peels of the fruits were cut into small pieces and dried for 24 h in a hot air oven (Memmert W., Büchenbach, Germany) at 100 °C. After drying, the samples were crushed into powder in a mechanical grinder (Moulinex, Bagnolet Cedex, France) and used in the diet formulation. The fine powder of PP peels was added to the basal diet at the levels of 10% and 20%. The ingredients of the diet were then mechanically mixed, pelletized, air-dried for 24 h at 25 °C, and finally stored at 4 °C until use. QU seeds (El Mashrq Company, Cairo, Egypt) were powdered and added to the basal diets at the above-mentioned levels (10% and 20%). The present study was a feeding trial based on the inclusion of these supplements into the diet to improve the growth rate and immune response of the other creatures [45,46].

### 2.3. Experimental Design

**Figure 1:**
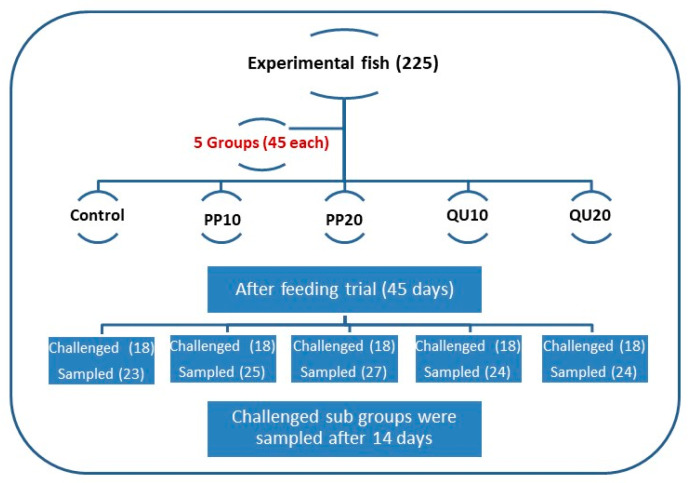
Schematic design of the experiment (groups, timing, and treatments). PP_10_: prickly pear fruit peel-supplemented group (10%), PP_20_: prickly pear fruit peel-supplemented group (20%), QU_10_: quinoa seeds—supplemented group (10%), QU_20_: quinoa seeds—supplemented group (20%).

The experimental fish were randomly allocated into five groups (*n* = 45 fish/group), and each group contained three replicates (15 fish/replicate). The first group (control) was fed on the basal diet without supplements. The second and third groups were fed on basal diets supplemented with 10% and 20% PP peel powder (PP_10_ and PP_20_-supplemented), respectively. The fourth and fifth groups were fed on basal diets supplemented with 10% and 20% QU seeds powder (QU_10_ and QU_20_-supplemented), respectively. The schematic design of the experiment is presented in Figure 1. The fish were fed twice daily (9:00 am and 3:00 pm) at a rate of 5% of the fish biomass throughout the experimental period (45 days). The fish biomass was recalculated every 14 days by weighing all the fish in each aquarium. The fish were euthanized using tricaine methanesulfonate, caught using a fishing net, and weighed as soon as possible after the capture using a digital household scale (0–2000 g). The daily mortality was recorded to determine the survival rate.

### 2.4. Growth Performance

The final body weight of the fish was obtained to estimate the gain in the body weight in all experimental groups (weight gain (g/fish) = (Wf) − (Wi)). The specific growth rate (SGR) was calculated according to the following equation of Jobling [47]: SGR (%/day) = 100 × ((ln Wf − ln Wi)/T), where “Wf” is the final weight in g, “Wi” is the initial weight in g, and “T” is the experimental duration in days.

### 2.5. Blood and Tissue Sampling

On the last day of the experimental period (45 days), the fish used in the sampling were euthanized using 250 mg/L of tricaine methanesulfonate. Two blood samples were collected from the caudal vein of each fish from all the experimental groups. The first set of blood samples (12 fish/group) were collected using sterile syringes without anticoagulant, held at room temperature (22 °C) for 6 h, and centrifuged at 1075× *g* for 20 min to separate the serum. The serum was then used for measuring the physiological biomarkers, digestive enzymes (lipase and amylase), and immunological markers. The second set of blood samples (6 fish/group) were collected in the EDTA-coated tubes to evaluate the hematological profile. The fish were killed by spinal cord sectioning, and the liver, intestine, and spleen tissues were excised. The liver samples were kept at −20 °C until analyzed for the determination of endogenous antioxidants and oxidative stress biomarkers. The collected spleen samples were quickly frozen in liquid nitrogen and then kept at −80 °C until the extraction of total RNA. A set of three tissue samples was fixed in 10% neutral-buffered formalin for histopathological and morphometric investigation.

### 2.6. Hematological Analysis

Red blood cell count, hemoglobin concentration, packed cell volume, mean corpuscular volume, mean corpuscular hemoglobin, mean corpuscular hemoglobin concentration (RBC, Hb, PCV, MCV, MCH, and MCHC), and total and differential leukocyte count in the blood samples were determined using an automatic cell counter (Hospitex Hema screen 18, Italy), following the method of Dacie and Lewis [48].

### 2.7. Serum Physiological Assays and Protein Profile

The liver enzymes aspartate aminotransferase (AST), alanine aminotransferase (ALT), and alkaline phosphatase (ALP) in the serum samples were assayed using Spinreact kits (Esteve De Bas, Spain) as reported in previous works [49,50,51]. Serum α-amylase and lipase activities were assayed using a rapid colorimetric kit from Spectrum Diagnostic Co. (Al-Obour, Egypt), according to Caraway [52] and Borlongan [53], following the manufacturer’s instructions. Total protein and albumin were quantified colorimetrically using BIOMED Diagnostic Egy. Chem. kits (Cairo, Egypt), according to Doumas et al. [54].

### 2.8. Antioxidant Status and Lipid Peroxidation Assays

Liver samples were rinsed with physiological saline and homogenized to estimate the activities of superoxide dismutase (SOD) and catalase (CAT), and the reduced glutathione (GSH) content, following the colorimetric method described by Nishikimi et al. [55], Aebi [56], and Beutler et al. [57], respectively. The lipid peroxidation marker malondialdehyde (MDA) was analyzed using the colorimetric method described by Ohkawa et al. [58].

### 2.9. Immune Response Assays

The lysozyme activity of serum was assayed spectrophotometrically based on the lysis of freeze-dried particles of *Micrococcus lysodeikticus,* according to the method of Ellis [59]. The nitric oxide (NO) content and the myeloperoxidase (MPO) activity in the serum were determined calorimetrically according to the assay described by Dymock [60] and Quade and Roth [61], respectively.

### 2.10. Transcriptional Analysis of Immune-Related Genes in the Spleen Tissue

The total RNA was extracted from the frozen spleen samples using the TRIzol reagent (easyREDTM, iNtRON Biotechnology, Seongnam-Si, Korea), following the manufacturer’s protocol. The first-strand cDNA was synthesized from the extracted RNA using a QuantiTect^®^ reverse transcription kit (Qiagen, Hilden, Germany) according to the manufacturer’s instructions. The sequences of the specific primers for the genes of interferon-*γ* (*IFN-γ*), Transforming growth factor-beta (*TGF-β*), and *EF-1α* (a housekeeping gene) are shown in Table 1. The qPCR analysis was performed in a Rotor-Gene Q instrument with a QuantiTect^®^ SYBR^®^ Green PCR kit (Qiagen, Germany) under the following conditions: 95 °C for 10 min, followed by 40 cycles of 95 °C for 15 s, 60 °C for 15 s, and 72 °C for 15 s. The melt-curve analysis was performed to validate the specificity of the PCR. The required relative fold change of the expression profile of the target genes was calculated using the comparative 2^−ΔΔCt^ method [62]. The ΔΔCt denotes the difference between the mean ΔCt of the supplemented group and the mean ΔCt of the control group, where ΔCt is the difference between the mean Ct of the gene of interest and the mean Ct of the internal control gene in each sample. Logarithmic transformation of fold-change values was performed before the statistical analysis.

### 2.11. Histological and Morphometric Methods

At the end of the feeding trial, the fixed tissue specimens (intestine, spleen, and liver) from each group were dehydrated in ascending grades of alcohol, cleared in xylene, embedded in paraffin, and sectioned at 5-µm thickness [63]. The sections were stained with hematoxylin and eosin (H&E), examined, and the images were captured using an AmScope microscope digital camera attached to an Olympus light microscope. The morphometric measurements of the intestine (villi length and width) and the number of goblet cells were obtained from the section images using the software ImageJ (version 1.51v; Research Services Branch, Bethesda, MD, USA). Changes in the splenic and hepatic parenchyma of fish were also analyzed. The morphometric measurements of the intestine were expressed in µm, and the counts of goblet cells were expressed as the number of goblet cells per unit surface area (mm^2^).

### 2.12. Aeromonas Sobria (A. sobria) Challenge Test

A pathogenic *A. sobria* strain was used for the challenge test. The strain was isolated from diseased Nile tilapia at the Fish Diseases and Management Department, Faculty of Veterinary Medicine, Zagazig University, Egypt. After the completion of the feeding trial of 45 days, the resistance of Nile tilapia to *A. sobria* was tested. Eighteen fish from each group were subjected to the challenge test, forming the challenged groups (control-challenged, PP_10_-challenged, PP_20_-challenged, QU_10_-challenged, and QU_20_-challenged). The fish were injected intraperitoneally with 0.1 mL cell suspension containing 1.5 × 10^7^ cells/mL (determined using a McFarland standard tube) [64]. The challenged fish were observed daily for two weeks to record the mortalities and/or clinical signs. The relative percentage survival (RPS) was calculated according to Amend [65] as follows: RPS = [1 − (% mortality in challenged fish/% mortality in control fish)] × 100.

On the 14th day following the bacterial challenge, the blood samples were obtained from the surviving fish (6 samples/group) and used for serum separation and estimation of protein profile, liver injury, and immune variables as described above. At necropsy, the tissue specimens (liver, intestine, and spleen) were fixed in 10% neutral-buffered formalin and processed for histopathological investigations. In addition, spleen samples were processed for the analysis of the gene expression profile of IFN-γ and TGF-β.

### 2.13. Statistical Analysis

The reported data were statistically analyzed by one-way analysis of variance (ANOVA) using the statistical program SPSS (version 16.0, SPSS Inc., Chicago, IL, USA). Tukey’s multiple comparison post hoc test was applied to compare the means of any two groups. The gene expression data were analyzed as fold change for six independent replicates of each treatment. Statistical significance was accepted at *p* < 0.05, and all the analyzed data were expressed as means ± SE (standard error).

## 3. Results

### 3.1. Survival Rate and Growth Performance in Nile Tilapia in Response to Dietary Supplementation with PP Peel or QU Seed

Supplementation of PP or QU to the diets increased the fish survival rate in the supplemented groups, particularly in PP_20_. A non-significant increase in the body weight gain was observed in the PP_10_, QU_10_, and QU_20_ groups, while a significant increase was observed in the PP_20_ group, compared to the control (Table 2). Moreover, SGR did not increase significantly in the fish fed with either QU- or PP-supplemented diets.

### 3.2. Hematological Variables of Nile Tilapia in Response to Dietary Supplementation with PP Peel or QU Seed

Increases in the RBC counts, Hb concentrations, and PCV levels were observed in the fish fed with the supplemented diets. The elevation in RBC and PCV values was significant in the PP_10_- and PP_20_-supplemented groups but insignificant in the QU-supplemented groups. Moreover, the highest significant increase in the Hb levels was exhibited by the PP_20_-supplemented group, followed by the PP_10_- and QU_20_-supplemented groups, while the QU_10_-supplemented group exhibited a non-significant increase (Table 3). The blood indices MCV, MCH, and MCHC, did not change significantly in any of the supplemented groups. The same trend was observed in the leukogram indices, with the highest counts of WBCs, lymphocytes, and monocytes recorded in the PP_20_-supplemented group, followed by the PP_10_- and QU_20_-supplemented groups. The supplementation of QU at the level of 10% did not affect these variables compared to the control group. Moreover, the heterophil count increased significantly in both fish groups fed on PP_10_- and PP_20_-supplemented diets and non-significantly in both QU-supplemented groups, compared to the control group. The basophil and eosinophil counts did not show a significant change in the supplemented groups versus the control (Table 3).

### 3.3. Physiological Biomarkers and Protein Profile in Nile Tilapia in Response to Dietary Supplementation with PP Peel or QU Seed

The levels of liver function indices in the experimental fish, including ALT, ALP, and AST, are shown in Table 3. A non-significant decline in the ALT activity was observed in the fish from the supplemented groups. In addition, AST and ALP levels showed a non-significant decline in response to supplementation with QU and PP_10_, while the decline in these indices was significant in the fish fed with 20% PP-supplemented diets (PP_20_). Moreover, there was a non-significant change in the levels of total protein, albumin, and globulin in all the supplemented fish groups. The serum lipase and amylase activities of Nile tilapia fed on different dietary levels of QU and PP were increased compared to the control. This increase was the highest in the fish fed with PP_20_–supplemented diet, followed by those fed with PP_10_- and QU-supplemented diets. The lowest increase in the activity of both the enzymes was observed in the fish fed with a 10% QU-supplemented diet.

### 3.4. Antioxidant and Oxidative Stress indices in Nile Tilapia in Response to Dietary Supplementation with PP or QU

**Figure 2:**
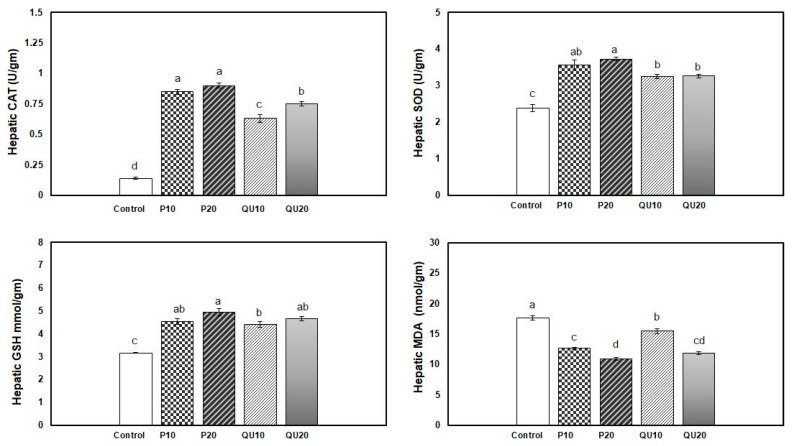
Effect of PP or QU supplementation at 10% and 20% for 45 days on the antioxidant status and oxidative stress variables in Nile tilapia. Values are expressed as mean ± SE (*n* = 6/group). The values that do not share a common superscript letter differ significantly at *p* < 0.05 by one-way ANOVA, followed by Tukey’s B post hoc test. PP_10_: prickly pear fruit peel-supplemented group (10%), PP_20_: prickly pear fruit peel-supplemented group (20%), QU_10_: quinoa seeds—supplemented group (10%), QU_20_: quinoa seeds—supplemented group (20%). Superoxide dismutase (SOD), catalase (CAT), reduced glutathione (GSH), and malondialdehyde (MDA).

The antioxidant levels in the fish fed on different levels of PP or QU showed a significant improvement, which was more conspicuous in the PP-supplemented groups. The highest activity of the CAT enzyme was observed in the PP_20_ and PP_10_-supplemented groups, followed by the QU_20_-supplemented group, while the lowest activity was observed in the QU_10_-supplemented fish (Figure 2). Moreover, the SOD level displayed a significant improvement in both PP-supplemented groups, followed by the QU-supplemented group. An improvement in the GSH level was recorded in all the PP- and QU-supplemented groups, which was similar among all the supplemented groups compared to the control fish. In contrast, the MDA level was significantly decreased in the supplemented groups, particularly in the fish fed with diets supplemented with PP and QU at the level of 20%, followed by those supplemented at the 10% level.

### 3.5. Indices of Immunological Response in Nile Tilapia Fed on Diets Supplemented with PP Peel or QU Seed

**Figure 3:**
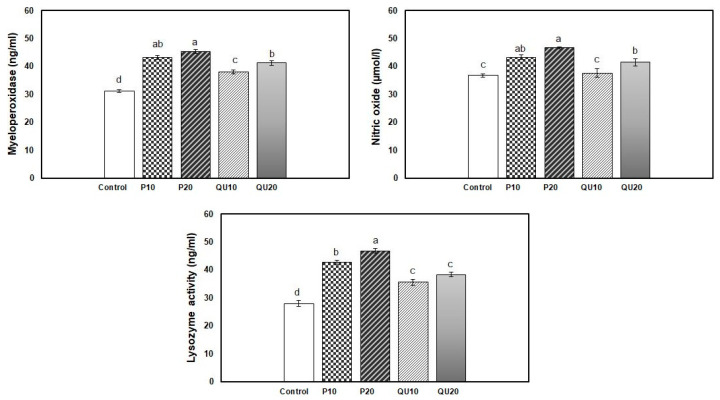
Effect of PP or QU supplementation at different levels (10% and 20%) for 45 days on the immune status variables in Nile tilapia. Values are expressed as mean ± SE (*n* = 6/group), and the values not sharing a common superscript letter differ significantly at *p* < 0.05 by one-way ANOVA followed by Tukey’s B post hoc test. PP_10_: prickly pear fruit peel-supplemented group (10%), PP_20_: prickly pear fruit peel-supplemented group (20%), QU_10_: quinoa seeds—supplemented group (10%), QU_20_: quinoa seeds—supplemented group (20%).

The 45-day supplementation of diets with PP or QU significantly enhanced the immunological response indices of Nile tilapia, including lysozyme activity, NO, and MPO levels, compared to the control group. A non-significant difference was observed in the MPO and NO levels in response to supplementation with PP, although the supplementation level of 20% PP showed a noteworthy increase, followed by the group supplemented with PP_10_. The improvement recorded in the QU_20_-supplemented group was statistically similar to that recorded in the PP_10_-supplemented group. The least improvement was recorded in the QU_10_–supplemented group. The highest lysozyme activity was observed in the PP_20_-supplemented group, compared to the control group. This was followed by the group receiving a diet supplemented with PP_10_, and finally, both groups supplemented with QU (QU_10_ and QU_20_) that showed similar lysozyme activities (Figure 3).

### 3.6. Gene expression Patterns of Immune-Encoding Genes in Nile Tilapia in Response to Dietary Supplementation with PP or QU

**Figure 4:**
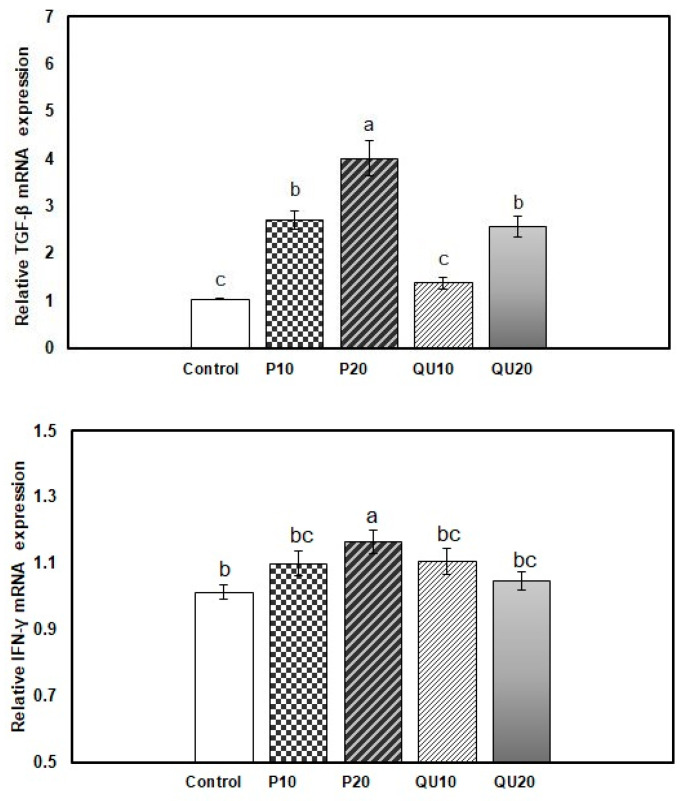
Relative expressions of the *TGF-β* and *IFN-γ* genes in the spleen tissue of Nile tilapia in response to PP or QU supplementation at 10% and 20% for 45 days. The expression abundance of the genes was normalized against the EF-1α internal control gene. Values are expressed as (mean ± SE, the values not sharing a common superscript letter (a, b, c) differ significantly at *p* < 0.05 by one-way ANOVA followed by Tukey’s B post hoc test. PP10: prickly pear fruit peel-supplemented group (10%), PP20: prickly pear fruit peel-supplemented group (20%), QU_10_: quinoa seeds- supplemented group (10%), QU_20_: quinoa seeds- supplemented group (20%).

In the spleen of fish from the supplemented groups, the transcription of the TGF-β gene was significantly upregulated upon the dietary supplementation with PP_20_, followed by PP_10_ and QU_20_ (4.01, 2.71, and 2.57-fold, respectively) (*p* = 0.001). The level of transcription of this gene was statistically similar in the PP_10_ and QU_20_-supplemented groups. The QU_10_-supplemented group showed a transcription level (of this gene) (1.38-fold) similar to that observed in the control fish (Figure 4). In contrast, the transcription of the IFN-γ gene was significantly upregulated in the fish fed with a PP_20_-supplemented diet (1.17-fold) (*p* = 0.031), but not in the other supplemented groups.

### 3.7. Histological Evidence in Nile Tilapia in Response to Dietary Supplementation with PP or QU

**Figure 5:**
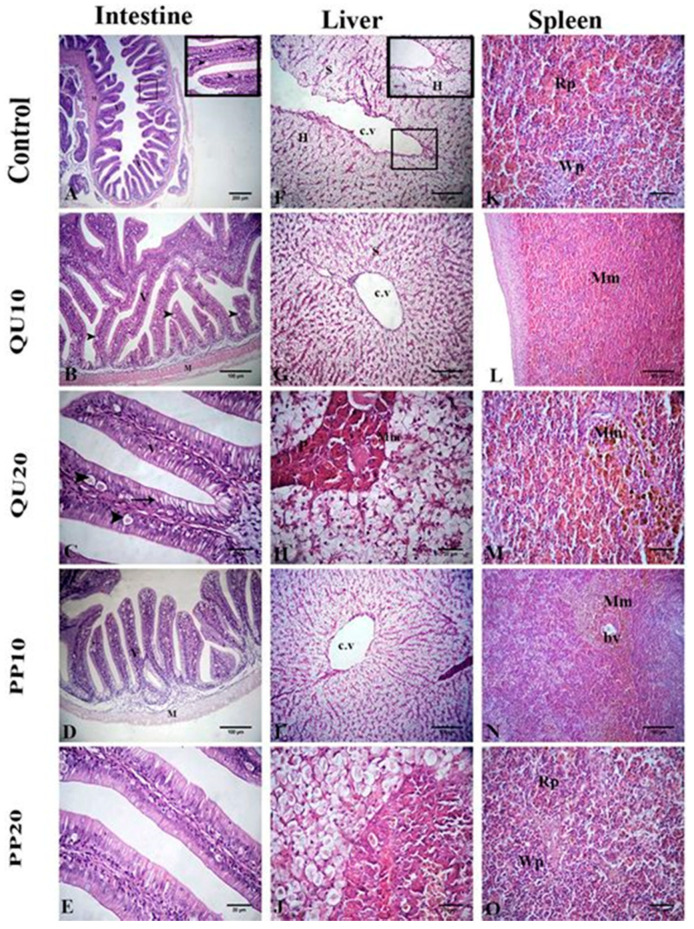
Representative photomicrographs of H&E-stained intestinal tissue sections of Nile tilapia (**A**–**E**): control group (**A**), QU_10_- (**B**), QU_20_- (**C**), PP_10_- (**D**), and PP_20_- (**E**) supplemented fish showing a normal intestinal wall, including columnar epithelium lining with the villi (thin arrow), large vacuoles (arrowheads), and thin propria submucosa (thick arrow) (villi (V) and musculosa (M)). Representative photomicrographs of H&E-stained liver-tissue sections of Nile tilapia (**F**–**J**): control group (**F**), QU_10_- (**G**), QU_20_- (**H**), PP_10_- (**I**), and PP_20_- (**J**) supplemented fish showing central vein (cv), portal vein (pv), normal hepatocytes (H), melanomacrophages (Mm), sinusoids (s), and pancreatic acini (p). Representative photomicrographs of H&E-stained spleen-tissue sections of Nile tilapia (**K**–**O**): control group (**K**), QU_10_- (**L**), QU_20_- (**M**), PP_10_- (**N**), and PP_20_- (**O**) supplemented fish showing a normal histological structure with red (Rp) and white pulps (Wp), along with melanomacrophage centers (Mm) around the blood vessel (bv).

The intestinal wall of the fish in the control group showed a normal histological structure. The tunica mucosa had villi that were lined with simple columnar epithelium with interspersed goblet cells. Large vacuoles were observed in the lining epithelium. The propria submucosa was constituted of loose connective tissue with no intestinal glands, and the tunica musculosa was formed of two layers of smooth muscle fibers. In the QU and PP-supplemented fish, the histological structure was similar to that in the control group, with an improvement in the intestinal mucosa. A significant increase in the width of the villi and the number of goblet cells, with a non-significant difference in the length of villi, was observed in the intestinal sections, particularly in the groups supplemented with 20% of PP or QU (Figure 5, Table 4).

Histological sections of the liver from all the experimental groups showed normal hepatic parenchyma that consisted of normal hepatocytes, central vein, sinusoids, melanomacrophage cells, and intrahepatic pancreatic tissue composed of peripheral secretory acini around the central portal vein. The splenic sections of all the experimental groups presented a normal structure with red and white pulps, along with normally distributed melanomacrophage centers (Mm) that were mainly associated with the vascular system (Figure 5).

### 3.8. Host Resistance Against A. sobria

#### 3.8.1. Mortality Rate, RPS, and Clinical Signs in the Surviving Nile Tilapia Supplemented with PP or QU and Challenged with *A. sobria*

After the challenge with *A. sobria*, the highest post-challenge RPS was recorded in the fish fed with PP supplementation at the level of 20%, followed by PP_10_- and QU_20_-supplemented fish. The lowest RPS was observed in the fish supplemented with QU at the level of 10%. In contrast, the highest mortality rate was recorded in the control group, while the PP_20_-supplemented fish showed the lowest rate of mortality (Table 5).

The challenged fish from different groups exhibited different degrees of escape reflex as follows: the fish receiving dietary supplementation with either 10% or 20% PP responded moderately to the external stimuli, while those fed with a diet supplemented with either level of QU were less active and showed a slight response to the external stimuli. Furthermore, lethargy, depression, and sluggish swimming movement, with a complete loss of escape reflex, were observed in the control group fish. The clinical symptoms observed in the infected fish were hemorrhages on different parts of the body, excessive mucus secretions on the skin and fins, distended abdomen, fin rot, and superficial-to-deep skin ulcers. Fish receiving only basal diet displayed severe signs of infection, including hemorrhage on the skin, large ulcers with hemorrhagic boundaries on the breast region, and smaller hemorrhagic regions on the caudal peduncle. Fish fed with the QU_10_-supplemented diet displayed severe unilateral cloudiness of the eye with large ulcers at the base of the pelvic fins. Fish fed with QU_20_-supplemented diets revealed moderate clinical signs in the form of petechial hemorrhage on the pectoral fin with redness of the mouth. The severity of disease symptoms reduced in the fish fed with a diet supplemented with PP at the level of 10%, showing mild disease symptoms in the form of petechial hemorrhage on the pectoral fin, with the accumulation of slime on the fins, particularly on the anal and caudal ones. Moreover, the fish receiving a PP_20_-supplemented diet appeared to have a better health condition, with the infected fish showing only a dark coloration of the body with a slight fin rot on the pectoral and caudal fins.

#### 3.8.2. Serum Physiological and Immunological Indices in Nile Tilapia Supplemented with PP or QU and Challenged with *A. sobria*

In the control-challenged group, the levels of total protein, albumin, and globulin were decreased in response to *A. sobria*, compared to the non-challenged fish of the control group. The decline in the total protein and albumin levels was significantly controlled in the challenged PP_20_ and QU_20_-supplemented groups. The groups supplemented with 10% PP or QU in their diets showed levels of these indices similar to those in the control-challenged group. Only the PP_20_-supplemented group showed significant control over the decline in the globulin levels. Such modulation in the protein profile indices was not observed in the control non-challenged fish. The levels of liver function indices were significantly elevated in the control-challenged fish, compared to the control non-challenged fish. This elevation was modulated in all the supplemented groups, with the challenged-PP_20_ group showing the highest modulation, followed by the challenged-QU_20_ and PP_10_ groups. The infected fish supplemented with QU_10_ showed the lowest modulation compared to the control-challenged group. Such modulation in the liver injury indices was not observed in the control non-challenged fish.

The immunological response indices exhibited a significant increase in the control-challenged fish, compared to the non-challenged ones from the control group. The lysozyme activity and MPO level showed a substantial increase in the challenged PP_20_-supplemented fish, followed by the challenged QU_20_-supplemented group, compared to the control-challenged group. The 10% dietary supplementation with QU and PP did not show a significant improvement in the lysozyme activity and MPO level. Furthermore, the NO levels were markedly modulated in the challenged PP_20_-supplemented group, followed by the groups supplemented with QU_20_ and PP_10_, compared with the control-challenged group. The NO level in the group supplemented with QU at the level of 10% was not modulated and was statistically similar to that observed in the control challenged fish (Table 5).

#### 3.8.3. Histological Evidence in Nile Tilapia Supplemented with PP or QU and Challenged with *A. sobria*

**Figure 6:**
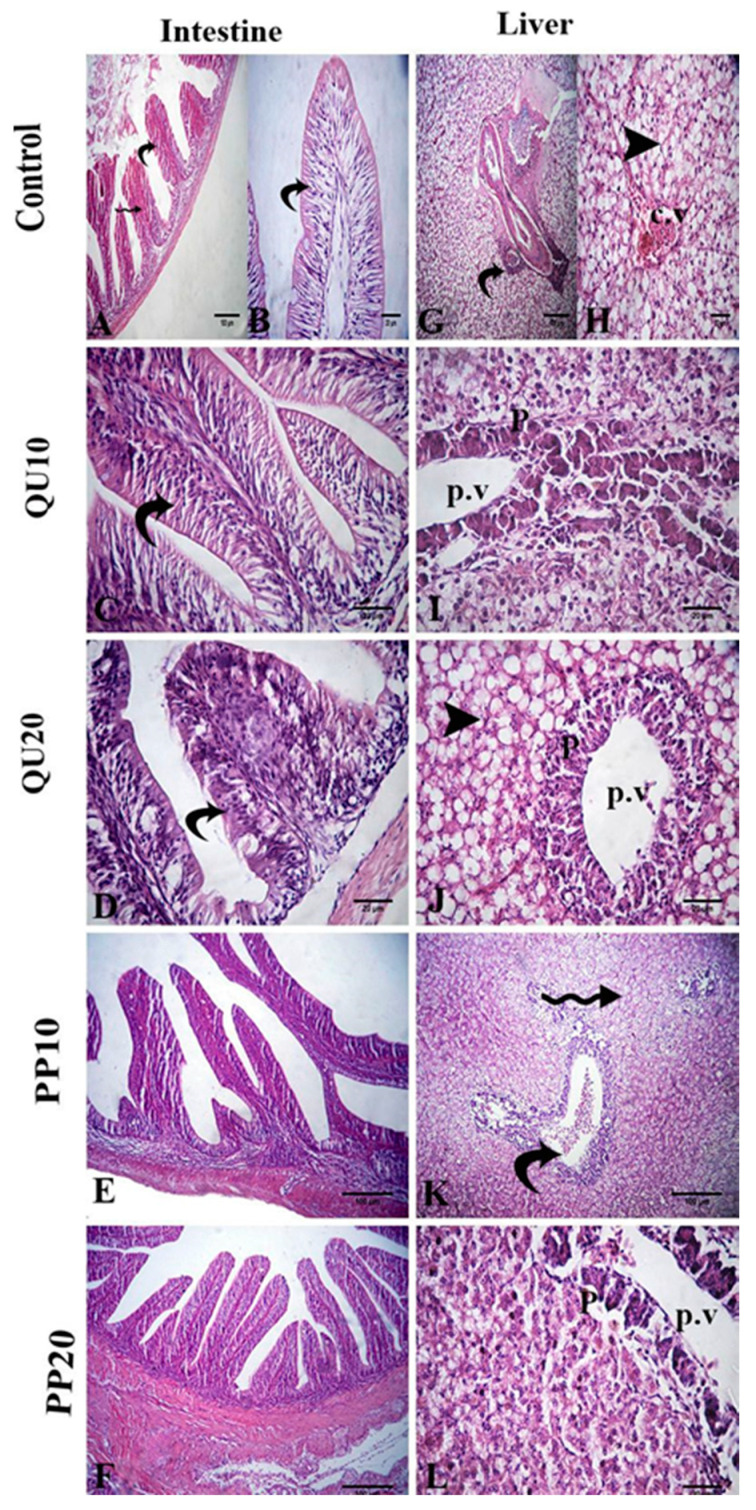
Photomicrographs of the H&E-stained intestinal tissue sections following the *A. sobria* challenge (**A**–**F**): *A. sobria*-challenged control fish (**A**,**B**) showing degeneration of epithelium (curved arrows), necrotized cells in the intestinal lumen (star), and hemorrhage in the submucosa (zigzag arrow). The challenged QU_10_-supplemented (**C**) and challenged QU_20_-supplemented fish (**D**) showing mild degeneration of the epithelium (curved arrows). The challenged PP_10_-supplemented (**E**) and challenged PP_20_-supplemented fish (**F**) showing normal intestinal villi. Photomicrographs of the H&E-stained liver tissue sections following the *A. sobria* challenge (**G**–**L**). *A. sobria*-challenged control fish (**G**,**H**) showing congested portal vein (curved arrow), congested central vein (cv), and fatty change (arrowhead). The challenged QU_10_-supplemented fish (**I**) showing mild necrosis of hepatocytes (arrow). The challenged QU_20_-supplemented fish (**J**) showing fatty change (arrowhead). The challenged PP_10_-supplemented fish (**K**) showing congested portal vein (curved arrow) and mild vacuolation of the hepatocytes (zigzag arrow). The challenged PP_20_-supplemented fish (**L**) showing mild necrosis of pancreatic acini (p).

The intestinal lumen of the challenged control fish showed degeneration in the columnar epithelium with necrotized cells. The submucosa showed hemorrhage and atrophy with epithelial erosion. In the challenged QU-supplemented groups, the villus epithelium showed nuclei, and cytoplasmic boundaries, which was an improvement compared to the control challenged group. In the challenged PP-supplemented groups, the improvement was more pronounced in the majority of the intestinal villi (Figure 6). A significant decrease in the length and width of the intestinal villi, as well as in the number of goblet cells, was observed in the control challenged group. This decrease was significantly modulated in the groups fed with the highest levels of the supplements (PP_20_ and QU_20_), followed by the PP_10_-supplemented group. In contrast, the QU_10_-supplemented group did not show a significant improvement in the histology compared to the control challenged fish (Figure 6, Table 4).

The liver of the control challenged fish showed a fatty change in the hepatocytes and congestion in the central vein, portal vein, and hepatic blood vessels. In addition, the hepatic sinusoids showed mild congestion. Restoration of these lesions was observed in the supplemented groups, recorded as only a mild fatty change and mild necrosis in the pancreatic tissue in both the challenged QU-supplemented groups. On the other hand, vacuolation of hepatocytes and congestion of the portal vein was observed in the challenged PP_10_-supplemented group. These changes were markedly decreased in the challenged PP_20_-supplemented group (Figure 6).

**Figure 7:**
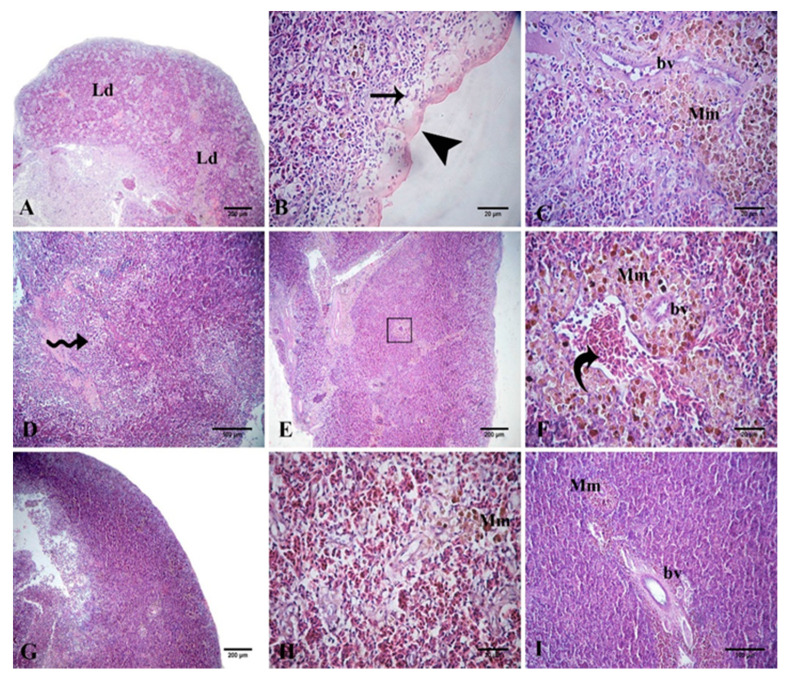
Representative photomicrographs of the H&E-stained Nile tilapia spleen-tissue sections following the *A. sobria* challenge. The *A. sobria*-challenged control fish (**A**–**C**) showing lymphoid depletion (Ld), necrosis (arrow) under the capsule (arrowhead), and activation of the melanomacrophage centers (Mm) around the blood vessel (bv). Challenged QU_10_-supplemented fish (**D**) showing diffuse infiltration of the inflammatory cells (zigzag arrows). Challenged QU_20_-supplemented fish (**E**) and its image at high magnification (**F**), showing the activation of the melanomacrophage centers (Mm) around the blood vessel (bv) and hemorrhage, with aggregates of the RBCs (curved arrow). Challenged PP_10_-supplemented fish (**G**,**H**) showing mild necrosis (**G**) and a mild increase in the melanomacrophage cells (Mm) (**H**). Challenged PP_20_-supplemented fish (**I**) showing a mild increase in the melanomacrophage cells (Mm) around the splenic blood vessels (bv).

The splenic tissue of the control challenged fish showed lymphoid depletion and subcapsular necrosis, which were associated with an increase in the melanomacrophage centers. In the QU_10_-supplemented group, necrosis and diffuse infiltration of the inflammatory cells were observed. The spleen tissue of the QU_20_-supplemented group showed mild lymphoid depletion, mild activation of the melanomacrophage centers, congestion, and aggregation of the RBCs. These changes were reduced in both the PP-supplemented groups, appearing in the form of aggregations of inflammatory cells with slight necrosis, along with a mild increase in the melanomacrophage centers (Figure 7).

#### 3.8.4. Expression Pattern of the Immune-Encoding Genes in Nile Tilapia Supplemented with PP or QU and Challenged with *A. sobria*

**Figure 8:**
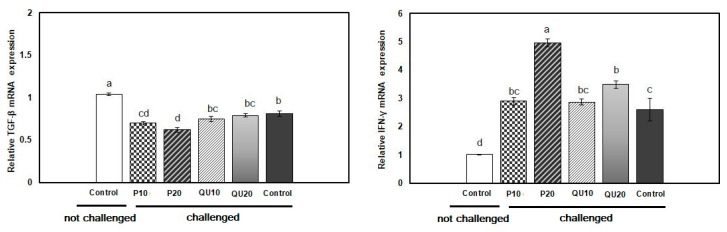
Relative expressions of *TGF-β* and *IFN-γ* mRNAs in the spleen tissue of *A. sobria*—challenged Nile tilapia in response to PP or QU supplementation at 10% and 20% for 45 days. The expression abundance of the genes was normalized against the *EF-1α* internal control gene. Values are expressed as (mean ± SE, the values not sharing a common superscript letter (a, b, c, d) differ significantly at *p* < 0.05 by one-way. PP10: prickly pear fruit peel-supplemented group (10%), PP20: prickly pear fruit peel-supplemented group (20%), QU_10_: quinoa seeds—supplemented group (10%), QU_20_: quinoa seeds—supplemented group (20%).

After the *A. sobria* challenge, the relative expression of TGF-β was significantly *(p* = 0.001) downregulated in the control challenged fish and in all the supplemented groups, compared to the control non-challenged fish. This downregulation was significant in the PP_10_ and PP_20_-supplemented fish (0.70- and 0.62-fold, respectively), compared to the control challenged fish. On the other hand, both QU-supplemented groups showed TGF-β expressions similar to those of the control challenged fish. The relative expression of IFN-γ in the spleen tissue was significantly *(p* = 0.001) upregulated in the PP_20_ and QU_20_-supplemented fish (4.96- and 3.49-fold, respectively), compared to both control challenged and control non-challenged fish. This upregulation was also recorded in the groups fed with 10% of either supplement, which was similar to the control challenged group but significantly different from the control non-challenged group (Figure 8).

## 4. Discussion

The present work evaluated the influence of PP peel and QU seeds as dietary supplements in fish feed on the immune status of Nile tilapia and its disease resistance against *A. sobria* infection, providing a novel perspective in aquaculture. Our results revealed that the dietary supplementation with PP and QU increased the survival rate in Nile tilapia through the protection of the hepatic tissue and the improvement of the digestive function, antioxidant status, and immunological responses. In addition, the supplementation improved the strength of the immune response of Nile tilapia against *A. sobria* infection, increasing their survival rate. This was evidenced by the modulation of immunological indices and the expressions of immune-encoding genes. Moreover, there was a restoration of the morphological structures of the intestine, liver, and spleen tissues, particularly at the supplementation level of 20%.

After 45 days, the body weight gain and survival rate were increased upon supplementation with PP, but not with QU. This was in line with the obvious improvement in the immune response in the PP-supplemented fish. Medicinal herbs mediate the growth of beneficial microbial colonies present in the digestive tract, leading to feed intake enhancement and weight gain in the supplemented fish [66]. This was confirmed in our study by the observed improvement in digestion, represented by a significant enhancement in the levels of digestive enzymes, an increase in the microvillus dimensions (height and width), and a healthy mucosal epithelium rich in goblet cells. Higher levels of total digestive enzymes were observed in the fish fed with PP-supplemented diets, which exhibited better growth performances compared to the control fish. The levels of digestive enzymes were positively correlated with growth promotion due to improvements in the intestinal secretions, nutrient digestion and absorption, and resistance to opportunistic indigenous bacteria [67]. Molina-Poveda et al. [68] stated that dietary supplementation with QU did not significantly influence the growth performance of shrimp (*Litopenaeus vannamei*), which could be attributed to the anti-nutritional factors in the plant that could influence the fish yield by reducing the utilization of proteins and consequently decreasing the growth rates.

Blood is a pathophysiological indicator of the entire body status and indicates the health status of organisms, including fish [69]. In this study, the recorded increase in the blood variables following the dietary supplementation with PP peel or QU seeds indicated the immune-stimulating effects and anti-infection properties of these supplements. Natural substances with antioxidant activity mainly do so by scavenging the reactive oxygen species (ROS) and stabilizing the red blood cell membrane [70]. Hence, the increase in the RBC count in this study may be related to the antioxidant effect of PP and QU seeds protecting the RBC membranes from hemolysis [71]. The increase in the RBC count may also be attributed to the progress of the formation of RBCs from the erythropoietic tissue in the presence of iron, vitamin A, B, C, and B12, which are essential for RBC production [72]. It is well known that PP is an important source of carbohydrates, polyunsaturated fatty acids, and natural antioxidants such as tocopherol, vitamin C, and vitamin E [73]. In addition, the QU seeds contain high levels of calcium, phosphorus, magnesium, iron, zinc, potassium, copper, and vitamin A, E, and B2 [74]. The Hb content in the blood of the fish was elevated in both PP- and QU_20_-supplemented groups, leading to an increase in oxygen transportation, consequently improving the wellbeing of fish and enhancing both immunity and growth [75].

The leukocyte count is an indicator of the health status of fish, as they play an important role in the nonspecific or innate immune response [76]. The number of lymphocytes produced reflects the strength and duration of the protection against diseased conditions [77]. The higher the number of WBCs, especially lymphocytes and other phagocytes (heterophils and monocytes), the better is the ability of an animal to perform well under stressful conditions and fight diseases [78]. Polysaccharides from different plants such as QU can enhance the transformation of lymphocytes and maintain their proliferation in immunosuppressed animals [79]. Our findings are supported by El-Neney et al. [46], who reported that the application of different levels of dried PP peels in the diet of rabbits for 14 weeks increased the levels of blood components (RBC and WBC counts and the Hb content). The increase in Hb and WBCs in the supplemented groups could be attributed mainly to the anti-inflammatory and antioxidant activities of the PP peel [80].

The bacterial challenge with *A. sobria* reduced the protein profile in the challenged fish, reflecting liver damage and protein loss, which was confirmed by the recorded rise in the liver enzymes and disruption of the cellular structure of the liver tissue. The reduction in the serum total protein may be due to the vascular leakage resulting from the increased permeability following the release of histamine or nonspecific proteolysis [81] or albumin losses from the external skin lesions, and due to reduced protein synthesis in the liver [82,83]. The protein profile was improved in the challenged fish supplemented with PP and QU, which was evident in the PP_10_-, PP_20_- and QU_20_-supplemented groups, reflecting the promotion of the humoral defense system of the fish. It is proposed that the observed modulation in the protein content may result from the restoration of protein synthesis in the liver tissue due to the hepatoprotective effects of both the supplements. Here, the control fish challenged with *A. sobria* showed a significant increase in the activities of AST, ALT, and ALP. The increase in these enzymes suggests the increased metabolic burden of the liver and damaged hepatocyte membranes [84]. Yu et al. [85] reported an increase in the activities of AST and ALT in *A. sobria*-infected mud loach, suggesting that these changes were directly related to cell membrane damage in the liver and kidney tissues. Supplementation with QU seed and PP peel at the level of 20% in the diet of the fish challenged with *A. sobria* showed a significant modulation in the activities of the enzymes AST, ALT, and ALP. This finding agrees with Saxena et al. [86], who reported that treatment with QU seed powder significantly reduced the activities of CCl_4_-induced hepatic enzymes in Swiss albino mice (*Mus musculus*). Moreover, pretreatment with PP extract significantly reduced the levels of serum hepatic enzymes elevated upon lithium exposure in Wistar rats (*Rattus norvegicus*) [16].

The hepatoprotective efficiency of natural products is mainly related to their high antioxidant contents, which restrict liver cell damage or death [87]. This was also confirmed by our results, with an increase in the antioxidant status of the supplemented-group fish. The hepatoprotective properties of QU seeds may be due to the presence of a high amount of antioxidants, phytochemicals, and flavonoids, such as quercetin, kaempferol, gallic acid, and phenolic acids, as well as fat-soluble vitamins, trace elements, fatty acids, and squalene, which could improve the antioxidant status of an organism [88,89]. Moreover, the antioxidant activity of QU seeds is highly correlated to non-phenolic compounds, which show strong free radical-scavenging activity [90]. PP is rich in flavonoids, including quercetin, taxifolin, and kaempferol, which are known for their ability to promote the formation and excretion of detoxified metabolites, which confers them with the liver-protective activity [91]. Moreover, the antioxidant activity is highly correlated with the number of phenolic constituents found in the PP extract since they act as good electron donors and may terminate the radical chain reaction by converting the free radicals into more stable products [92]. Morán-Ramos et al. [93] reported that the beneficial effect of the dietary consumption of PP in an obese Zucker rat model was associated with a decrease in the hepatic oxidative stress and hepatic injury biomarkers, owing to the direct interaction between the antioxidant molecules present in PP and the ROS. Recently, Blando et al. [94] indicated that the extract of PP cladodes exhibited in vitro and in vivo antioxidant activities with strong anti-hemolytic activity, as well as a selective inhibition efficacy against potentially pathogenic bacteria. These activities were attributed to the highly active phenolic components of PP, such as p-hydroxybenzoic acid derivatives.

A significant increase in the antioxidant status (SOD, CAT activity, and GSH content), with a significant decrease in the MDA level, was noticed in the fish supplemented with QU, indicating the antioxidant potency of both the supplements. Zhai and Liu [95] reported that PP increased the antioxidant potential of hepato-pancreas in Nile tilapia. In addition, Ben Saad et al. [16] reported that the administration of PP extract had a strong hepatoprotective effect on the oxidative lithium-mediated damage in rat models, which could be attributed to the antioxidant activity of the active constituents such as phenolics, flavonoids, and polysaccharides. Avila-Nava et al. [96] reported that PP exhibited both in vitro and in vivo antioxidant activities; therefore, its consumption protected the body from oxidative stress. This may be due to the existence of polyphenolic compounds, vitamin C and E, β-carotene, and total carotenoids. Moreover, Pasko et al. [20] reported that the antioxidative system was more efficient when the QU seeds were incorporated into the diet, as they could restrict the oxidative stress by decreasing the generation of free radicals during the pathological state via a reduction in lipid peroxidation and improvement of the antioxidant capacity of blood and various organs.

A compound shows immunostimulatory efficacy when it augments the immune response of an animal challenged with a pathogen, thereby enabling the animal to survive following the challenge. Hence, the immune-stimulating abilities of PP and QU in fish were tested in the present study. The control challenged fish showed typical clinical signs of *A. sobria* infection, including skin ulcers, fin erosion, eye infections, and hemorrhagic septicemia. Moreover, the fish responded to bacterial infection through changes in serum biochemistry, protein profile, immunology, and tissue histopathology. Our findings indicated that dietary supplementation with PP and QU significantly improved the disease resistance, represented by a significant reduction in the mortalities and an increase in the RPS compared to the fish fed with a diet without the supplements. The high RSP recorded in this study could be attributed to the beneficial effects of these supplements on the immune response, as well as to their antioxidant properties [97]. Aeromonas infection is highly correlated with the presence of oxidative stress [98]. Our findings indicated that the inclusion of PP and QU in the diet improved the antioxidant defense of fish and enhanced the components of innate immunity. This improved antioxidant status following the dietary supplementation may confer a higher capacity for disease prevention to the fish. Moreover, the observed fish tolerance to the challenge indicated that dietary PP and QU had a significant capability of immunomodulation, represented by the enhancement of nonspecific immune parameters in the pre-challenge period, including lysozyme activity, NO, and MPO, as well as by the modulation of the expressions of immune-related genes.

In this study, dietary supplementation with QU seeds and PP peels promoted the activities of lysozyme and MPO and increased the NO content in Nile tilapia, indicating an immunostimulatory potential via the induction of lysis of the bacterial cell walls, stimulation of the phagocytosis of bacteria, and improvement of the innate immune response [99]. The elevation of lysozyme and NO levels has been previously demonstrated in various fish species supplemented with immunostimulants, such as spirulina (*Spirulina platensis*), *thyme* (*Thymus vulgaris*), and guava (*Psidium guajava*) leaves [64,85,100,101,102]. This may be attributed to the bioactive constituents of these supplements, which possibly induce the leukocytes and increase the amount of lysozyme synthesized per cell [103]. The increased NO content in different dietary supplements supported the role of NO in enhancing the nonspecific immune response. Here, the improved innate immune response could be due to the high flavonoid contents in PP and QU, which stimulate the leucocytes and the process of phagocytosis [104].

Moreover, both the supplements improved the resistance against *A. sobria* infection owing to their bactericidal effect. Sun et al. [105] reported that the oral pathogenic bacteria could be restrained by the use of crude saponins extracted from QU, which caused severe damage to the bacteria. This was evidenced by the debasement of the cell wall, followed by disruption of the cytoplasmic membrane and membrane proteins, resulting in the leakage of the cell contents [106]. In addition, the PP peel was reported to exhibit antibacterial potential owing to various bioactive constituents, such as sterols, flavonoids, tannins, phenolics, and alkaloids, which inhibited microbial growth through their ability to inactivate the microbial enzymes, adhesion, and cell envelope-transport proteins, as well as by forming a complex with the polysaccharides [71,107]. The antimicrobial activity of the PP fruit pulp or peel could be attributed to ferulic, piscidic, caffeic, eucomic, and other phenolic constituents, which have been reported to be more inhibitory and toxic to microorganisms [108].

Our study also demonstrated the anti-inflammatory properties of PP and QU, evidenced by the upregulation of TGF-β in the pre-challenge period. On the other hand, following the challenge, TGF-β was downregulated, indicating an increase in the pro-inflammatory cytokines, suggesting the immune-modulating effect in the infected fish. Normally, the rise in TGF-β inhibits the proliferation and differentiation of B and T cells and antagonizes the pro-inflammatory cytokines (e.g., IL-1β, TNFα, and IFN-γ) [109]. This was confirmed by the recorded upregulation of IFN- γ in response to the challenge, which was more obvious in the groups supplemented with high levels of PP and QU. This could indicate a defense mechanism in which the fish interpret *A. sobria* as a threat, as evidenced by the improvement in the immune response to resist its infection. On the other hand, moderate transcription of the pro-inflammatory cytokines is effective in the maintenance of the immunological balance and improvement of resistance against infection. The observed upregulation in the expression pattern of the pro-inflammatory cytokine (IFN-γ) in the infected Nile tilapia was in line with the findings of Moustafa et al. [98], who demonstrated upregulation in the expression of pro-inflammatory immune response-related genes (*TNF-α* and *IL-1β*) following infection with *A. hydrophila*. Hu et al. and Yao et al. [110,111] confirmed the immunoregulatory activity of the polysaccharides isolated from QU seeds causing improvement in the proliferation of the RAW264.7 macrophage. The other benefits of these supplements include the regulation of immune function by enhancing the immune response through the activation of signaling pathways and upregulation of the expression of cytokines (IL-6, IFN-γ, and IFN-ɑ), improvement of the IgM and lysozyme content in the serum, and the enhancement of the phagocytic function of mononuclear macrophages [112].

The kidney head, spleen, and liver are involved in fish immunity as these organs incorporate the immune cells within their tissues [113]. In the current study, the addition of PP and QU to the diet of fish diminished the pathological changes associated with *A. sobria* infection in all the examined organs, with PP supplementation showing a more obvious effect. Therefore, the provision of a good diet appears to be an important factor for the maintenance of cellular homeostasis and histoarchitecture in fish [114]. Similar pathological changes were reported in the Nile tilapia infected with *A. hydrophila* [115] and Japanese flounder (*Paralichthys olivaceus*) fish infected with *Edwardsiella tarda* [116]. Moreover, Ostaszewska et al. [117] demonstrated a restoration in the hepatocyte architecture of silver bream (*Vimba vimba*) fish supplemented with natural feed additives. In addition, Owatari et al. [118] reported that silymarin significantly diminished the pathological lesions in the spleen and liver of *O. niloticus* challenged with *Streptococcus agalactiae*. Moustafa et al. [98] reported that Fenugreek seed powder restored the intestine, spleen, liver, and kidney tissues of *O. niloticus* infected with *A. hydrophila.*

The detrimental impact of bacterial infections on the organism’s tissues is partly associated with the promotion of oxidative stress [119]. The flavonoids present in the plants provide a beneficial antioxidant effect through their capability of scavenging the ROS and consequently reducing their harmful effects [120]. The Mm aggregations or centers in the spleen tissue are associated with the immune response through the phagocytosis of foreign particles [121]. In line with our results, an increase in the Mm number was observed in *O. niloticus* fed with a diet supplemented with *Echinacea purpurea* [122] and in farmed sea bass (*Dicentrarchus labrax* L.) fish fed with polyphenol-enriched diet [123]. Post-challenge, the intestine samples from the control groups exhibited marked necrosis and degeneration of the intestinal epithelium, the severity of which was reduced in the challenged groups fed with PP and QU-supplemented diets. Similar pathological alterations were observed when the infected Nile tilapia were fed with a diet supplemented with Fenugreek seed powder as an immunostimulant [98].

## 5. Conclusions

In conclusion, our results indicated that the Nile tilapia fed with diets containing different levels of PP peel and QU seeds showed effective antioxidant activity and immune response, especially at the 20% level, which enabled the Nile tilapia to resist the *A. sobria* infection. This performance of the fish was attributed to the positive effects of PP and QU on the immune function, antioxidant capacity, hepatoprotective potency, tissue architecture restoration, and disease resistance of the fish against *A. sobria* challenge. Hence, the use of these herbal plants as immunostimulants represents a cost-effective alternative in Nile tilapia aquaculture.

## Figures and Tables

**Table 1:** Primer sequences used for real-time qPCR analysis of immune-related genes.

Gene	Forward	Amplicon Size (bp)	Accession No.
IFN-γ	F: 5′-AGC ACA ACG TAG CTT TCC CT-3′ R: 5′-TAA ACA GGG CAA ACA GGT CA-3′	132	XM_003460533.2
TGF-β	F: 5′-GTTTGAACTTCGGCGGTACTG-3′ R: 5′-TCCTGCTCATAGTCCCAGAGA-3′	80	XM_003459454.2
EF-1α	F: 5′-TGATCTACAAGTGCGGAGGAA-3′ R: 5′-GGAGCCCTTTCCCATCTCA-3′	80	AB075952.1

Interferon-γ (IFN-γ), transforming growth factor-beta (TGF-β), and elongation factor 1 alpha (EF-1α).

**Table 2:** Effect of PP or QU supplementation for 45 days on the survival rate and growth performance of Nile tilapia.

Parameters	Experimental Groups				
	Control	PP_10_	PP_20_	QU_10_	QU_20_
Survival rate					
Surviving fish/group (No./group)	41/45	43/45	45/45	42/45	42/45
Survival rate (%)	91.11%	95.50%	100%	93.33%	93.33%
Bodyweight					
Initial body weight (g)	23.12 ± 1.04	23.35 ± 0.48	23.59 ± 0.16	22.77 ± 0.83	23.61 ± 0.78
Final body weight (g)	32.40 ± 1.28 ^b^	35.33 ± 0.38 ^ab^	36.00 ± 0.36 ^a^	34.17 ± 0.54 ^ab^	34.00 ± 0.46 ^ab^
Body weight gain (g)	9.28 ± 0.32 ^b^	11.98 ± 0.86 ^ab^	12.41 ± 0.42 ^a^	11.40 ± 0.67 ^ab^	10.38 ± 0.67 ^ab^
Specific growth rate (%/day)	0.76 ± 0.04	0.92 ± 0.07	0.94 ± 0.03	0.88 ± 0.06	0.81 ± 0.06

Values are mean± SE for the replicate/group (*n*= 3 replicate); the values not sharing a common superscript letter differ significantly at *p* < 0.05 by one-way ANOVA followed by Tukey’s B post hoc test. PP_10_: prickly pear fruit peel-supplemented group (10%), PP_20_: prickly pear fruit peel-supplemented group (20%). QU_10_: quinoa seeds—supplemented group (10%), QU_20_: quinoa seeds—supplemented group (20%).

**Table 3:** Effect of PP or QU supplementation for 45 days on the hematologic and serum physiological variables in Nile tilapia.

Parameters	Experimental Groups				
	Control	PP_10_	PP_20_	QU_10_	QU_20_
Erythrogram					
RBCs (10^6^/µL)	2.82 ± 0.11 ^c^	3.47 ± 0.10 ^ab^	3.63 ± 0.11 ^a^	2.91 ± 0.13 ^c^	3.08 ± 0.11 ^bc^
Hb (g/dL)	8.27 ± 0.21 ^c^	9.73 ± 0.12 ^a^	10.17 ± 0.20 ^a^	8.55 ± 0.13 ^bc^	9.02 ± 0.12 ^b^
PCV (%)	29.16 ± 1.29 ^c^	36.39 ± 1.53 ^ab^	38.72 ± 1.08 ^a^	30.1 ± 1.26 ^c^	32.71 ± 1.20 ^bc^
MCV (fL)	103.4 ± 0.81	104.87 ± 1.27	106.67 ± 0.96	103.43 ± 0.63	106.2 ± 0.86
MCH (pg)	29.33 ± 0.65	28.05 ± 0.66	27.97 ± 0.45	29.38 ± 0.79	29.29 ± 0.64
MCHC (%)	28.37 ± 0.65	26.74 ± 1.07	26.25 ± 0.35	28.4 ± 0.78	27.59 ± 0.62
Leukogram					
WBCs (10^3^/µL)	6.51 ± 0.16 ^c^	8.54 ± 0.25 ^ab^	9.01 ± 0.02 ^a^	7.06 ± 0.16 ^c^	7.87 ± 0.19 ^b^
Lymphocytes (10^3^/µL)	3.89 ± 0.10 ^c^	5.28 ± 0.11 ^a^	5.55 ± 0.16 ^a^	4.23 ± 0.12 ^c^	4.8 ± 0.13 ^b^
Heterophils (10^3^/µL)	1.89 ± 0.076 ^c^	2.40 ± 0.14 ^ab^	2.59 ± 0.10 ^a^	2.07 ± 0.10 ^bc^	2.26 ± 0.10 ^abc^
Monocytes (10^3^/µL)	0.46 ± 0.01 ^d^	0.58 ± 0.02 ^ab^	0.62 ± 0.02 ^a^	0.51 ± 0.01 ^cd^	0.54 ± 0.01 ^bc^
Eosinophils (10^3^/µL)	0.20 ± 0.01	0.21 ± 0.009	0.19 ± 0.01	0.18 ± 0.02	0.22 ± 0.01
Basophils(10^3^/µL)	0.065 ± 0.001	0.061 ± 0.001	0.064 ± 0.001	0.066 ± 0.002	0.067 ± 0.002
Liver function tests					
ALT (U/L)	24.02 ± 0.26	23.21 ± 0.19	22.85 ± 0.60	23.77 ± 0.44	22.73 ± 0.41
AST(U/L)	34.20 ± 0.32 ^a^	34.15 ± 0.28 ^a^	32.77 ± 0.19 ^b^	33.90 ± 0.30 ^ab^	33.20 ± 1.00 ^ab^
ALP(U/L)	49.00 ± 0.26 ^a^	48.02 ± 0.22 ^a^	46.93 ± 0.47 ^b^	48.98 ± 0.62 ^ab^	47.73 ± 0.18 ^ab^
Protein profile					
Total protein (g/dL)	6.76 ± 0.10	6.86 ± 0.13	6.73 ± 0.19	6.68 ± 0.10	6.93 ± 0.14
Albumin (A)	3.62 ± 0.12	3.82 ± 0.13	3.62 ± 0.15	3.57 ± 0.11	3.84 ± 0.15
Globulin (G)	3.15 ± 0.03	3.04 ± 0.02	3.11 ± 0.07	3.11 ± 0.02	3.10 ± 0.03
Digestive enzymes					
Amylase (U/L)	40.67 ± 0.61 ^d^	55.33 ± 0.71 ^b^	63.33 ± 1.33 ^a^	48.50 ± 0.99 ^c^	58.33 ± 0.92 ^b^
Lipase (U/L)	25.50 ± 1.64 ^e^	46.83 ± 1.49 ^b^	52.00 ± 1.37 ^a^	30.50 ± 1.23 ^d^	41.16 ± 1.05 ^c^

Values are mean ± SE (*n* = 6/group); the values not sharing a common superscript letter differ significantly at *p* < 0.05 by One-way ANOVA followed by Tukey’s B post hoc test. PP_10_: prickly pear fruit peel-supplemented group (10%), PP_20_: prickly pear fruit peel-supplemented group (20%). QU_10_: quinoa seeds—supplemented group (10%), QU_20_: quinoa seeds—supplemented group (20%). Red blood cell count (RBCs), hemoglobin concentration (Hb), packed cell volume (PCV), mean corpuscular volume (MCV), mean corpuscular hemoglobin (MCH), mean corpuscular hemoglobin concentration (MCHC), White blood cell count (WBCs), aspartate aminotransferases (AST), alanine aminotransferase (ALT), and alkaline phosphatase (ALP).

**Table 4:** Effect of the challenge with *A. sobria* on mortality rate, RPS, liver function and immune status of *O. niloticus* fish supplemented with PP or QU in different levels (10% and 20%).

Parameters	Experimental Groups				
	Control	PP_10_	PP_20_	QU_10_	QU_20_
Pre challenge					
Intestinal villi length (μm)	300.06 ± 7.77	310.36 ± 17.68	342.51 ± 29.00	298.87 ± 13.75	314.42 ± 9.56
Intestinal villi width (μm)	37.37 ± 1.94 ^c^	46.34 ± 1.39 ^b^	52.96 ± 1.39 ^a^	40.31 ± 1.71 ^c^	46.92 ± 1.84 ^b^
Goblet cell count (cell/ mm^2^)	20.83 ± 1.25 ^b^	26.00 ± 1.06 ^b^	32.17 ± 2.18 ^a^	21.67 ± 1.20 ^b^	24.50 ± 1.18 ^b^
	**Control (Challenged)**	**PP_10 (Challenged)_**	**PP_20 (Challenged)_**	**QU_10 (Challenged)_**	**QU_20 (Challenged)_**
Post challenge					
Intestinal villi length (μm)	208.20 ± 12.44 ^c^	289.83 ± 9.46 ^a^	291.83 ± 14.15 ^a^	223.34 ± 18.28 ^bc^	277.88 ± 24.37 ^ab^
Intestinal villi width (μm)	34.35 ± 0.97 ^c^	38.33 ± 1.59 ^bc^	47.45 ± 2.61 ^a^	38.38 ± 1.51 ^bc^	43.00 ± 2.15 ^ab^
Goblet cell count (cell/ mm^2^)	16.33 ± 1.28 ^b^	22.00 ± 1.93 ^a^	26.00 ± 1.46 ^a^	16.83 ± 0.70 ^b^	22.83 ± 1.01 ^a^

Values are mean ± SE (*n* = 6/group), values are not sharing a common superscript letter (a, b, c) differ significantly at *p* < 0.05. One-way ANOVA followed by Tukey’s B post hoc test. PP10: Prickly pear fruit peel-supplemented group (10%), PP20: Prickly pear fruit peel-supplemented group (20%). QU10: Quinoa seeds -supplemented group (10%), QU20: Quinoa seeds -supplemented group (20%).

**Table 5:** Effect of the challenge with *A. sobria* on mortality rate, RPS, liver function and immune status of Nile tilapia supplemented with PP or QU for 45 days.

Parameters	Experimental Groups					
	Control (Non-Challenged)	Control (Challenged)	PP_10_ (Challenged)	PP_20_ (Challenged)	QU_10_ (Challenged)	QU_20_ (Challenged)
Mortality (number and rate)	-	9/18 (50%)	3/18 (16.66%)	2/18 (11.11%)	5/18 (27.77%)	3/18 (16.66%)
RPS	-	0.00%	66.66%	77.77%	44.44%	66.66%
Liver function tests						
ALT (U/L)	24.02 ± 0.26 ^f^	56.13 ± 0.60 ^a^	31.63 ± 0.49 ^d^	28.9 ± 0.36 ^e^	36.17 ± 0.90 ^b^	33.77 ± 0.37 ^c^
AST(U/L)	34.20 ± 0.32 ^d^	57.02 ± 0.82 ^a^	39.50 ± 1.36 ^c^	35.77 ± 0.77 ^d^	44.8 ± 0.54 ^b^	42.28 ± 0.70 ^bc^
ALP(U/L)	49.00 ± 0.26 ^f^	113.33 ± 1.63 ^a^	73.17 ± 1.47 ^c^	57.83 ± 1.47 ^e^	81.92 ± 1.80 ^b^	67.37 ± 1.54 ^d^
Protein profile						
Total protein (g/dL)	6.76 ± 0.10 ^a^	4.34 ± 0.12 ^d^	5.07 ± 0.10 ^c^	5.85 ± 0.10 ^b^	4.58 ± 0.08 ^d^	5.09 ± 0.10 ^c^
Albumin (A)	3.62 ± 0.12 ^a^	2.40 ± 0.10 ^c^	2.81 ± 0.14 ^bc^	3.04 ± 0.19 ^b^	2.61 ± 0.08 ^bc^	2.97 ± 0.14 ^b^
Globulin (G)	3.15 ± 0.03 ^a^	1.93 ± 0.12 ^b^	2.25 ± 0.08 ^b^	2.81 ± 0.20 ^a^	1.97 ± 0.07 ^b^	1.95 ± 0.11 ^b^
Immune status						
Lysozyme activity (ng/mL)	28.00 ± 1.06 ^d^	36.00 ± 0. 82 ^c^	38.67 ± 1.05 ^c^	50.33 ± 0.76 ^a^	37.16 ± 0.95 ^c^	42.33 ± 1.05 ^b^
Nitric oxide (µmol/L)	36.83 ± 0.60 ^d^	43.30 ± 0.88 ^c^	49.73 ± 1.06 ^b^	55.06 ± 1.47 ^a^	45.73 ± 0.89 ^bc^	48.82 ± 1.56 ^b^
Myeloperoxidase (ng/mL)	31.18 ± 0.48 ^d^	40.17 ± 0.77 ^c^	41.77 ± 0.92 ^c^	53.42 ± 1.13 ^a^	42.10 ± 2.08 ^c^	48.67 ± 0.88 ^b^

Values are mean ± SE (*n* = 6/group) the values not sharing a common superscript letter differ significantly at *p* < 0.05 by one-way ANOVA followed by Tukey’s B post hoc test. PP_10_: prickly pear fruit peel-supplemented group (10%), PP_20_: prickly pear fruit peel-supplemented group (20%); QU_10_: quinoa seeds—supplemented group (10%), QU_20_: quinoa seeds—supplemented group (20%); relative percentage survival (RPS), aspartate aminotransferases (AST), alanine aminotransferase (ALT), and alkaline phosphatase (ALP).

## Supplementary Materials

The following are available online at https://www.mdpi.com/2076-2615/10/12/2266/s1, Table S1: Formulation and calculated composition analysis of the basal and experimental diets fed to Nile tilapia.
