# Supplementary material for: Influence of Feeding Quinoa (Chenopodium quinoa) Seeds and Prickly Pear Fruit (Opuntia ficus indica) Peel on the Immune Response and Resistance to Aeromonas sobria Infection in Nile Tilapia (Oreochromis niloticus)
Source: Animals (Basel). 2020 Dec 1;10(12):2266. doi: 10.3390/ani10122266 (PMC7760620; doi:10.3390/ani10122266)
Supplement: Supplementary file 1 [file animals-10-02266-s001.pdf]

animals

MDPI

# Supplementary Materials: Influence of Feeding Quinoa (*Chenopodium quinoa*) Seeds and Prickly Pear Fruit (*Opuntia ficus indica*) Peel on the Immune Response and Resistance to *Aeromonas sobria* Infection in Nile Tilapia (*Oreochromis niloticus*)

**Shaimaa A.A. Ahmed [1], Ghada I. Abd El-Rahman [2], Amany Behairy [3], Rasha R. Beheiry [4], Basma M. Hendam [5], Faisal M. Alsubaie [6] and Samah R. Khalil [7,*]**

1 Department of Fish Diseases and Management, Faculty of Veterinary Medicine, Zagazig University, Zagazig 44511, Egypt; shaimaakarim2020@gmail.com
2 Department of Clinical Pathology, Faculty of Veterinary Medicine, Zagazig University, Zagazig 44511, Egypt; gana660@gmail.com
3 Department of Physiology, Faculty of Veterinary Medicine, Zagazig University, Zagazig 44511, Egypt; amanybehairy25688@gmail.com
4 Department of Histology and Cytology, Faculty of Veterinary Medicine, Zagazig University, Zagazig 44511, Egypt; rasharagab2006@yahoo.com
5 Department of Husbandry and Development of Animal Wealth, Faculty of Veterinary Medicine, Mansoura University, Mansoura 35511, Mansoura, Egypt; d.basma.gentic@gmail.com
6 Department of Genome, Central Veterinary Laboratory, Riyadh 11454, Saudi Arabia; iamfaisal77@gmail.com
7 Department of Forensic Medicine and Toxicology, Faculty of Veterinary Medicine, Zagazig University Zagazig 44511, Egypt
* Correspondence: resamah@zu.edu.eg; Tel.: + 201033966915

Received: 5 November 2020; Accepted: 29 November 2020; Published: 1 December 2020

**Table S1.** Formulation and calculated composition analysis of the basal and experimental diets fed to *O. niloticus* fish.

| Items | g /kg diet* | | | | |
|---|---|---|---|---|---|
| | Control | PP10 | PP20 | QU10 | QU20 |
| Ingredient | | | | | |
| Yellow corn | 210 | 210 | 210 | 210 | 210 |
| Soybean meal 48% CP | 200 | 200 | 200 | 200 | 200 |
| Fish meal | 150 | 150 | 150 | 150 | 150 |
| Corn gluten 60% CP | 130 | 130 | 130 | 130 | 130 |
| Rice bran | 110 | 110 | 110 | 110 | 110 |
| Wheat middlings | 150 | 150 | 150 | 150 | 150 |
| Premix-Min** | 10 | 10 | 10 | 10 | 10 |
| Premix-Vit*** | 10 | 10 | 10 | 10 | 10 |
| Corn oil | 30 | 30 | 30 | 30 | 30 |
| Total | 1000 | 1000 | 1000 | 1000 | 1000 |
| Calculated composition (%) | | | | | |
| Crude protein | 32.05 | 27.00 | 29.00 | 31.00 | 32.00 |
| Lipid | 4.55 | 7.00 | 9.00 | 6.00 | 6.00 |
| Crude fiber | 4.24 | 5.57 | 5.70 | 3.86 | 5.35 |
| Ash | 7.30 | 7.60 | 6.30 | 8.30 | 6.60 |
| Nitrogen free extract**** | 51.85 | 52.83 | 50.00 | 50.84 | 50.05 |

*Control: (basal diet without any additives), $PP_{10}$: (900 g basal diet+100 g Prickly pear fruit peel), $PP_{20}$ (800 g basal diet+200 g Prickly pear fruit peel), $QU_{10}$: (900 g basal diet+100 g Quinoa seeds), and $QU_{20}$: (800 g basal diet+200 g Quinoa seeds). **Composition of mineral premix kg−1: manganese, 53 g; zinc, 40 g; iron, 20 g; copper, 2.7 g; iodine, 0.34 g; selenium, 70 mg; cobalt, 70 mg and calcium carbonate as carrier up to 1 kg. ***Composition of vitamin premix kg−1: vitamin A, 8,000,000 IU; vitamin D3, 2,000,000 IU; vitamin E, 7,000 mg; vitamin K3, 1,500 mg; vitamin B1, 700 mg; vitamin B2, 3,500 mg; vitamin B6, 1,000 mg; vitamin B12, 7 mg; biotin, 50 mg; folic acid, 700 mg; nicotinic, 20,000 mg; pantothenic acid, 7,000 mg. ****Nitrogen free extract = 100 − (crude protein + Crude lipids + ash + crude fiber).
